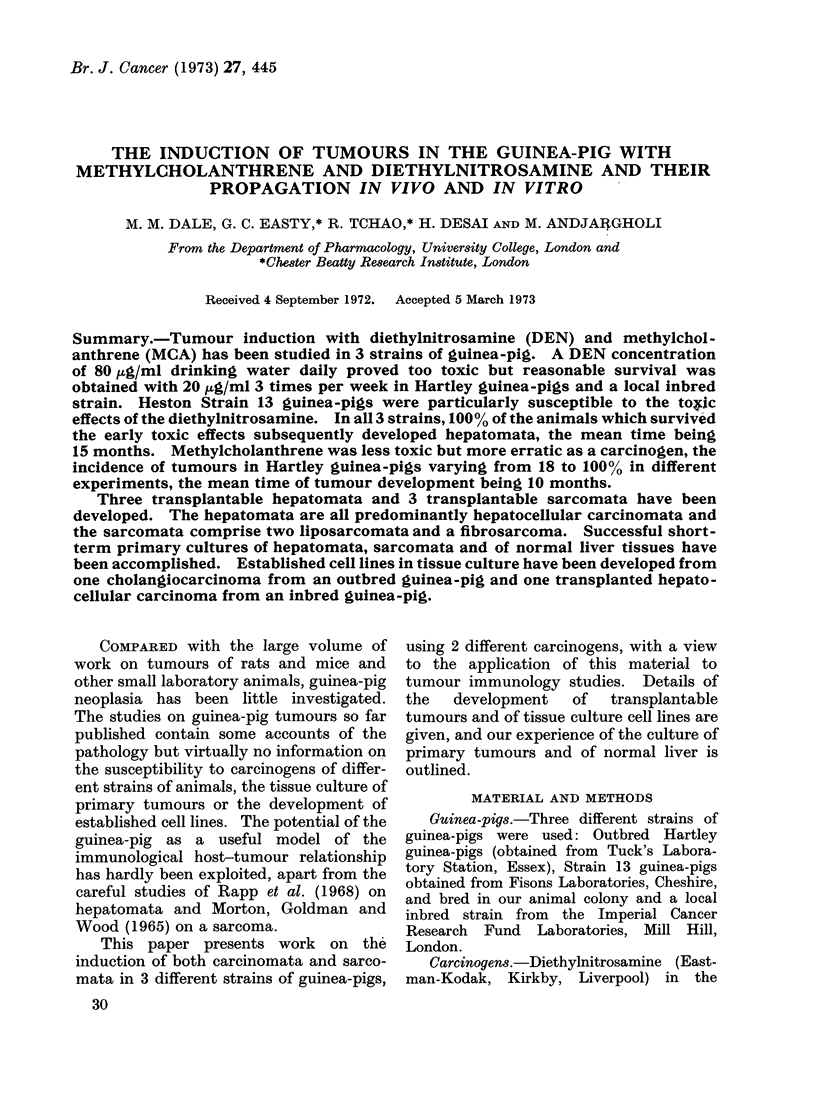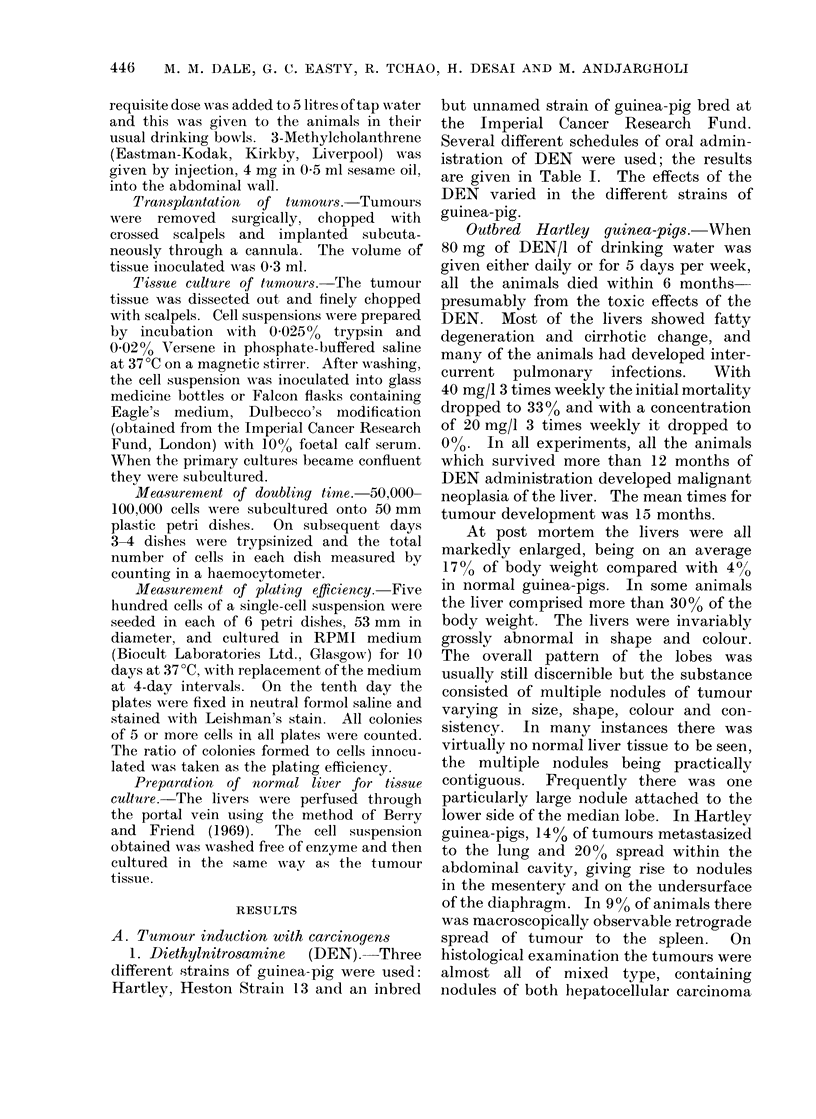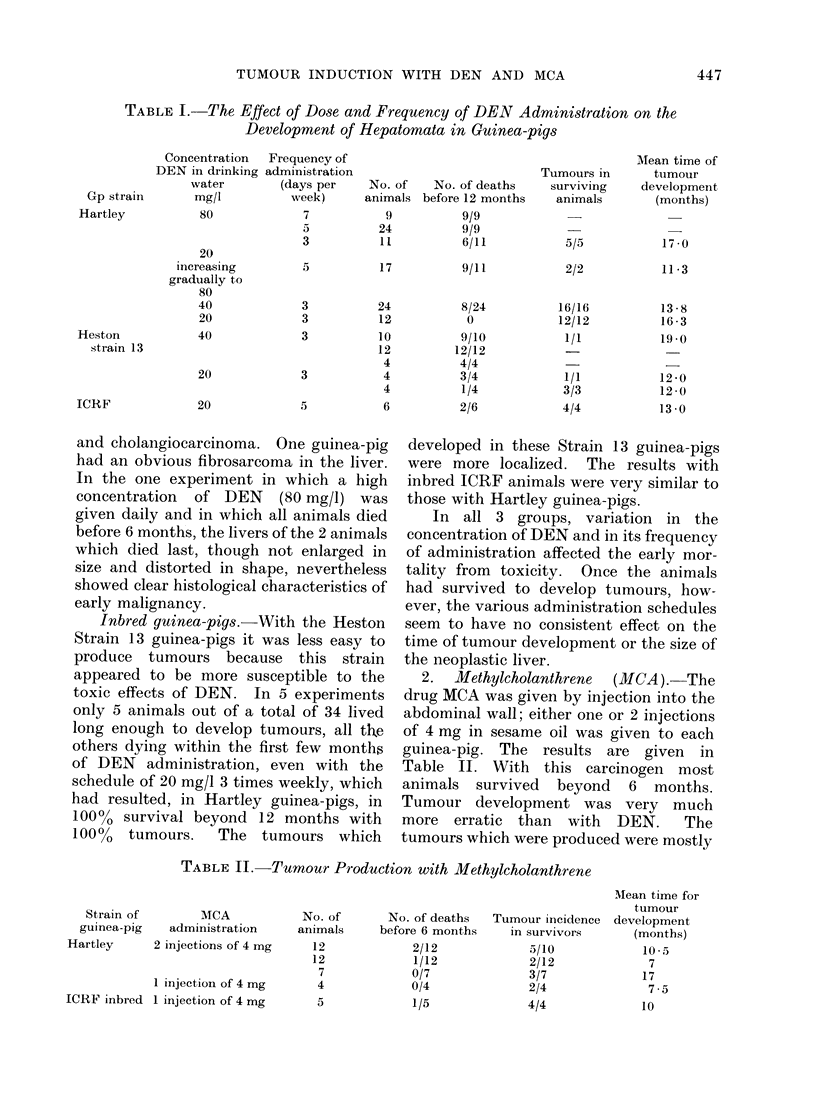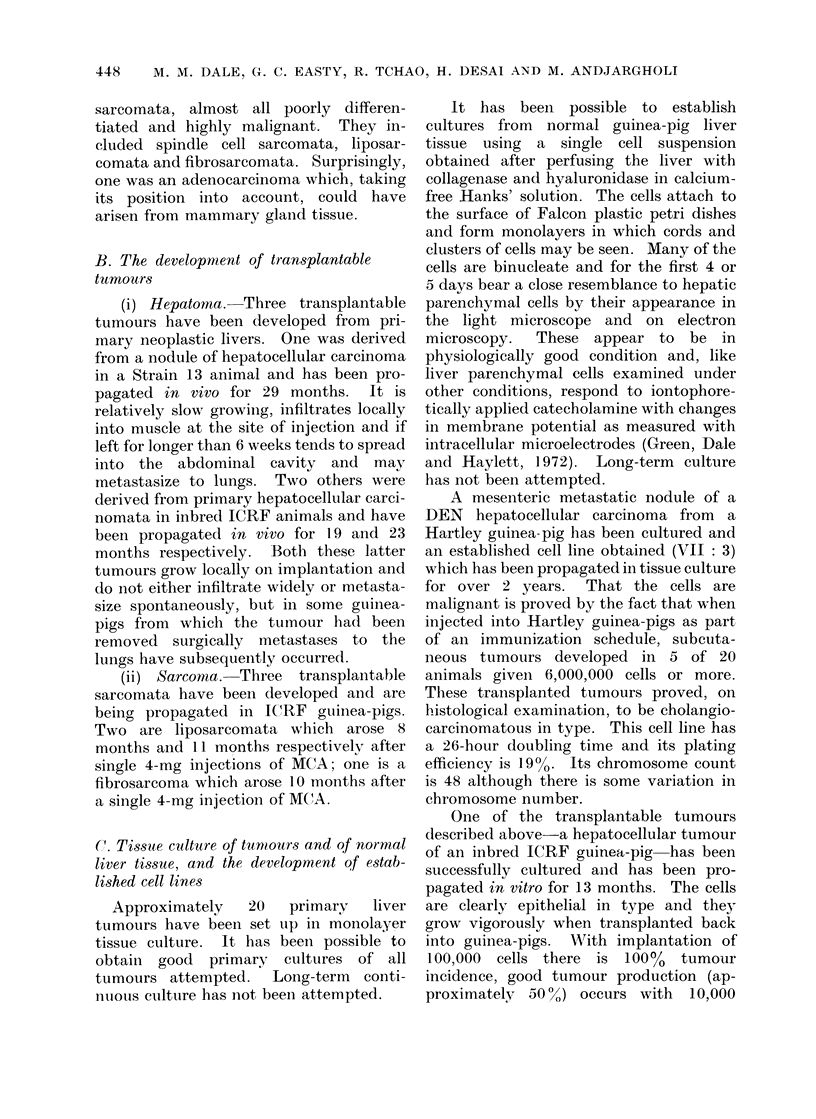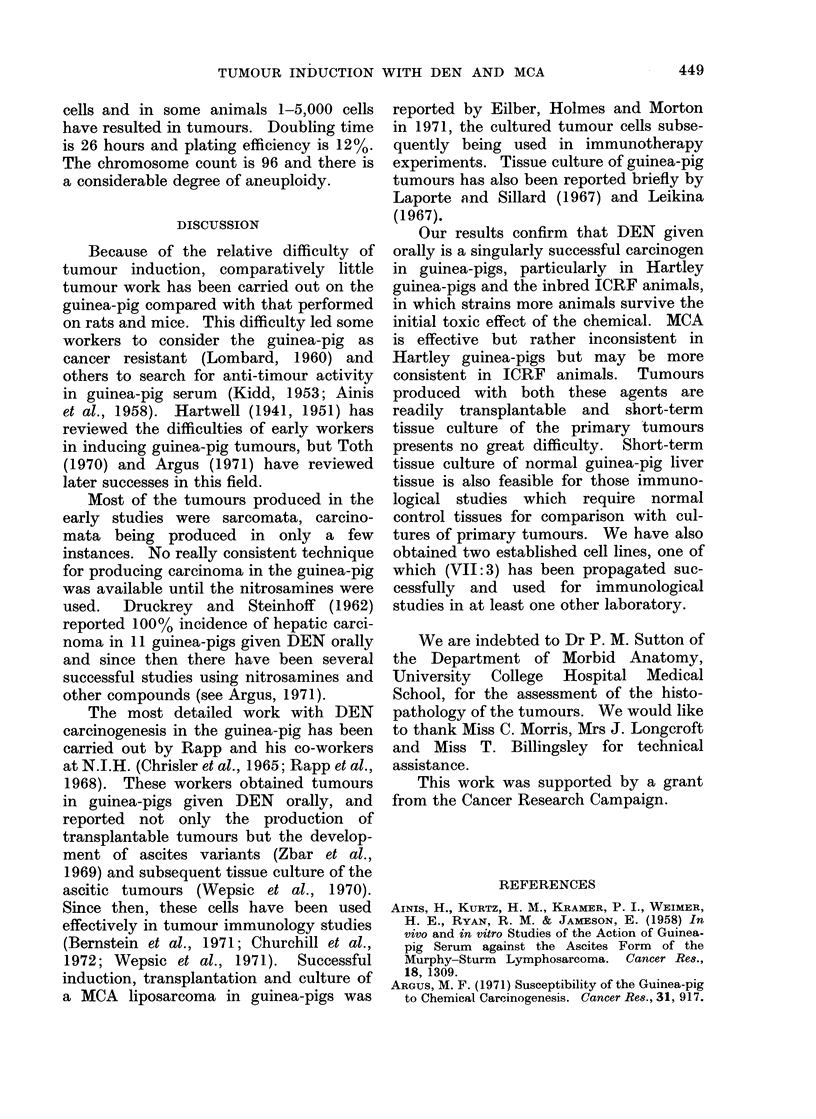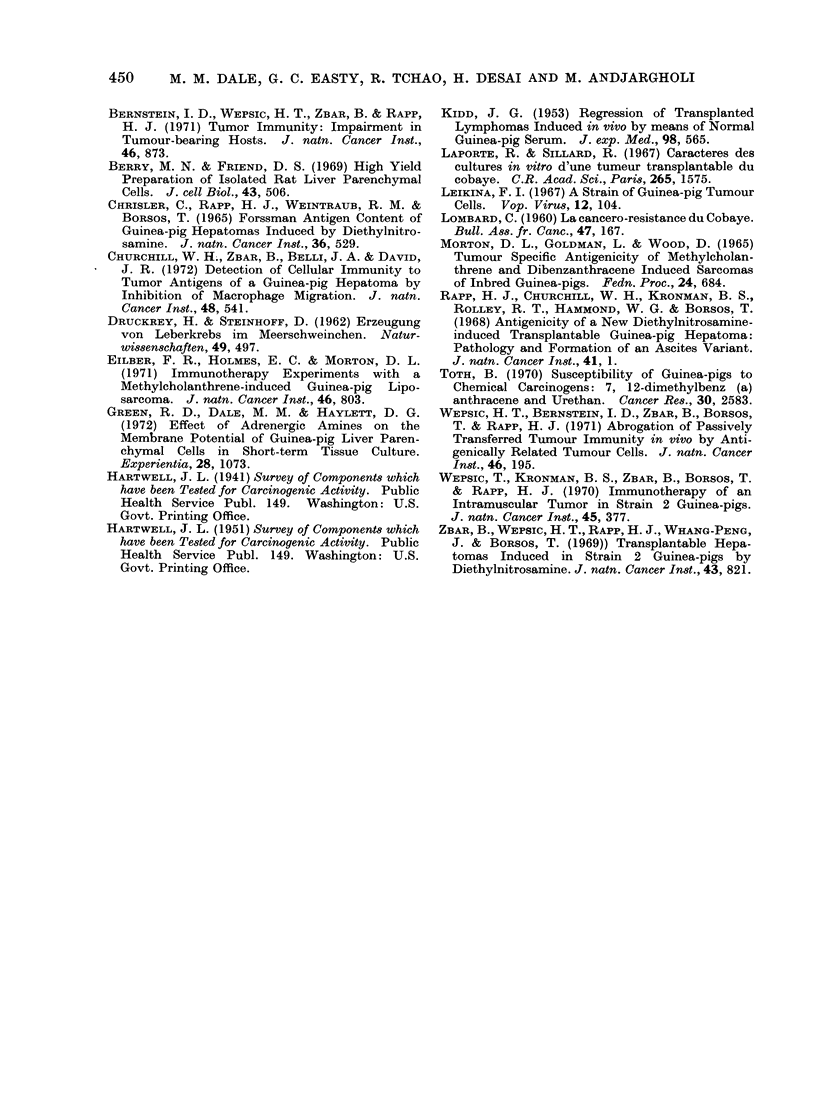# The Induction of Tumours in the Guinea-pig with Methylcholanthrene and Diethylnitrosamine and their Propagation In Vivo and in vitro

**DOI:** 10.1038/bjc.1973.56

**Published:** 1973-06

**Authors:** M. M. Dale, G. C. Easty, R. Tchao, H. Desai, M. Andjargholi

## Abstract

Tumour induction with diethylnitrosamine (DEN) and methylcholanthrene (MCA) has been studied in 3 strains of guinea-pig. A DEN concentration of 80 μg/ml drinking water daily proved too toxic but reasonable survival was obtained with 20 μg/ml 3 times per week in Hartley guinea-pigs and a local inbred strain. Heston Strain 13 guinea-pigs were particularly susceptible to the toxic effects of the diethylnitrosamine. In all3strains, 100% of the animals which survived the early toxic effects subsequently developed hepatomata, the mean time being 15 months. Methylcholanthrene was less toxic but more erratic as a carcinogen, the incidence of tumours in Hartley guinea-pigs varying from 18 to 100% in different experiments, the mean time of tumour development being 10 months.

Three transplantable hepatomata and 3 transplantable sarcomata have been developed. The hepatomata are all predominantly hepatocellular carcinomata and the sarcomata comprise two liposarcomata and a fibrosarcoma. Successful shortterm primary cultures of hepatomata, sarcomata and of normal liver tissues have been accomplished. Established cell lines in tissue culture have been developed from one cholangiocarcinoma from an outbred guinea-pig and one transplanted hepatocellular carcinoma from an inbred guinea-pig.


*Br. J. Cancer* (1973) **27**, 445

### THE INDUCTION OF TUMOURS IN THE GUINEA-PIG WITH METHYLCHOLANTHRENE AND DIETHYLNITROSAMINE AND THEIR PROPAGATION *IN VIVO* AND *IN VITRO*

M. M. DALE, G. C. EASTY,* R. TCHAO,* H. DESAI AND M. ANDJARGHOLI

*From the Department of Pharmacology, University College, London and*
**Chester Beatty Research Institute, London*

Received 4 September 1972. Accepted 5 March 1973

**Summary.—Tumour induction with diethylnitrosamine (DEN) and methylcholanthrene (MCA) has been studied in 3 strains of guinea-pig. A DEN concentration of 80 μg/ml drinking water daily proved too toxic but reasonable survival was obtained with 20 μg/ml 3 times per week in Hartley guinea-pigs and a local inbred strain. Heston Strain 13 guinea-pigs were particularly susceptible to the toxic effects of the diethylnitrosamine. In all 3 strains, 100% of the animals which survived the early toxic effects subsequently developed hepatomata, the mean time being 15 months. Methylcholanthrene was less toxic but more erratic as a carcinogen, the incidence of tumours in Hartley guinea-pigs varying from 18 to 100% in different experiments, the mean time of tumour development being 10 months.**

**Three transplantable hepatomata and 3 transplantable sarcomata have been developed. The hepatomata are all predominantly hepatocellular carcinomata and the sarcomata comprise two liposarcomata and a fibrosarcoma. Successful short-term primary cultures of hepatomata, sarcomata and of normal liver tissues have been accomplished. Established cell lines in tissue culture have been developed from one cholangiocarcinoma from an outbred guinea-pig and one transplanted hepatocellular carcinoma from an inbred guinea-pig.**

COMPARED with the large volume of work on tumours of rats and mice and other small laboratory animals, guinea-pig neoplasia has been little investigated. The studies on guinea-pig tumours so far published contain some accounts of the pathology but virtually no information on the susceptibility to carcinogens of different strains of animals, the tissue culture of primary tumours or the development of established cell lines. The potential of the guinea-pig as a useful model of the immunological host–tumour relationship has hardly been exploited, apart from the careful studies of Rapp *et al.* (1968) on hepatomata and Morton, Goldman and Wood (1965) on a sarcoma.

This paper presents work on the induction of both carcinomata and sarcomata in 3 different strains of guinea-pigs, using 2 different carcinogens, with a view to the application of this material to tumour immunology studies. Details of the development of transplantable tumours and of tissue culture cell lines are given, and our experience of the culture of primary tumours and of normal liver is outlined.

### MATERIAL AND METHODS

*Guinea-pigs.*—Three different strains of guinea-pigs were used: Outbred Hartley guinea-pigs (obtained from Tuck's Laboratory Station, Essex), Strain 13 guinea-pigs obtained from Fisons Laboratories, Cheshire, and bred in our animal colony and a local inbred strain from the Imperial Cancer Research Fund Laboratories, Mill Hill, London.

*Carcinogens.*—Diethylnitrosamine (Eastman-Kodak, Kirkby, Liverpool) in the requisite dose was added to 5 litres of tap water and this was given to the animals in their usual drinking bowls. 3-Methylcholanthrene (Eastman-Kodak, Kirkby, Liverpool) was given by injection, 4 mg in 0·5 ml sesame oil, into the abdominal wall.

*Transplantation of tumours.*—Tumours were removed surgically, chopped with crossed scalpels and implanted subcutaneously through a cannula. The volume of tissue inoculated was 0·3 ml.

*Tissue culture of tumours.*—The tumour tissue was dissected out and finely chopped with scalpels. Cell suspensions were prepared by incubation with 0·025% trypsin and 0·02% Versene in phosphate-buffered saline at 37 °C on a magnetic stirrer. After washing, the cell suspension was inoculated into glass medicine bottles or Falcon flasks containing Eagle's medium, Dulbecco's modification (obtained from the Imperial Cancer Research Fund, London) with 10% foetal calf serum. When the primary cultures became confluent they were subcultured.

*Measurement of doubling time.*—50,000–100,000 cells were subcultured onto 50 mm plastic petri dishes. On subsequent days 3–4 dishes were trypsinized and the total number of cells in each dish measured by counting in a haemocytometer.

*Measurement of plating efficiency.*—Five hundred cells of a single-cell suspension were seeded in each of 6 petri dishes, 53 mm in diameter, and cultured in RPMI medium (Biocult Laboratories Ltd., Glasgow) for 10 days at 37 °C, with replacement of the medium at 4-day intervals. On the tenth day the plates were fixed in neutral formol saline and stained with Leishman's stain. All colonies of 5 or more cells in all plates were counted. The ratio of colonies formed to cells innoculated was taken as the plating efficiency.

*Preparation of normal liver for tissue culture.*—The livers were perfused through the portal vein using the method of Berry and Friend (1969). The cell suspension obtained was washed free of enzyme and then cultured in the same way as the tumour tissue.

### RESULTS

### *A. Tumour induction with carcinogens*

1. *Diethylnitrosamine* (DEN).—Three different strains of guinea-pig were used: Hartley, Heston Strain 13 and an inbred but unnamed strain of guinea-pig bred at the Imperial Cancer Research Fund. Several different schedules of oral administration of DEN were used; the results are given in Table I. The effects of the DEN varied in the different strains of guinea-pig.

*Outbred Hartley guinea-pigs.*—When 80 mg of DEN/l of drinking water was given either daily or for 5 days per week, all the animals died within 6 months—presumably from the toxic effects of the DEN. Most of the livers showed fatty degeneration and cirrhotic change, and many of the animals had developed intercurrent pulmonary infections. With 40 mg/l 3 times weekly the initial mortality dropped to 33% and with a concentration of 20 mg/l 3 times weekly it dropped to 0%. In all experiments, all the animals which survived more than 12 months of DEN administration developed malignant neoplasia of the liver. The mean times for tumour development was 15 months.

At post mortem the livers were all markedly enlarged, being on an average 17% of body weight compared with 4% in normal guinea-pigs. In some animals the liver comprised more than 30% of the body weight. The livers were invariably grossly abnormal in shape and colour. The overall pattern of the lobes was usually still discernible but the substance consisted of multiple nodules of tumour varying in size, shape, colour and consistency. In many instances there was virtually no normal liver tissue to be seen, the multiple nodules being practically contiguous. Frequently there was one particularly large nodule attached to the lower side of the median lobe. In Hartley guinea-pigs, 14% of tumours metastasized to the lung and 20% spread within the abdominal cavity, giving rise to nodules in the mesentery and on the undersurface of the diaphragm. In 9% of animals there was macroscopically observable retrograde spread of tumour to the spleen. On histological examination the tumours were almost all of mixed type, containing nodules of both hepatocellular carcinoma

TABLE I.—*The Effect of Dose and Frequency of DEN Administration on the Development of Hepatomata in Guinea-pigs*

| Gp strain | Concentration DEN in drinking water mg/l | Frequency of administration (days per week) | No. of animals | No. of deaths before 12 months | Tumours in surviving animals | Mean time of tumour development (months) |
|---|---|---|---|---|---|---|
| Hartley | 80 | 7 | 9 | 9/9 | — | — |
| | | 5 | 24 | 9/9 | — | — |
| | | 3 | 11 | 6/11 | 5/5 | 17·0 |
| | 20 increasing gradually to 80 | 5 | 17 | 9/11 | 2/2 | 11·3 |
| | 40 | 3 | 24 | 8/24 | 16/16 | 13·8 |
| | 20 | 3 | 12 | 0 | 12/12 | 16·3 |
| Heston strain 13 | 40 | 3 | 10 | 9/10 | 1/1 | 19·0 |
| | | | 12 | 12/12 | — | — |
| | | | 4 | 4/4 | — | — |
| | 20 | 3 | 4 | 3/4 | 1/1 | 12·0 |
| | | | 4 | 1/4 | 3/3 | 12·0 |
| ICRF | 20 | 5 | 6 | 2/6 | 4/4 | 13·0 |

and cholangiocarcinoma. One guinea-pig had an obvious fibrosarcoma in the liver. In the one experiment in which a high concentration of DEN (80 mg/l) was given daily and in which all animals died before 6 months, the livers of the 2 animals which died last, though not enlarged in size and distorted in shape, nevertheless showed clear histological characteristics of early malignancy.

*Inbred guinea-pigs.*—With the Heston Strain 13 guinea-pigs it was less easy to produce tumours because this strain appeared to be more susceptible to the toxic effects of DEN. In 5 experiments only 5 animals out of a total of 34 lived long enough to develop tumours, all the others dying within the first few months of DEN administration, even with the schedule of 20 mg/l 3 times weekly, which had resulted, in Hartley guinea-pigs, in 100% survival beyond 12 months with 100% tumours. The tumours which developed in these Strain 13 guinea-pigs were more localized. The results with inbred ICRF animals were very similar to those with Hartley guinea-pigs.

In all 3 groups, variation in the concentration of DEN and in its frequency of administration affected the early mortality from toxicity. Once the animals had survived to develop tumours, however, the various administration schedules seem to have no consistent effect on the time of tumour development or the size of the neoplastic liver.

2. *Methylcholanthrene* (*MCA*).—The drug MCA was given by injection into the abdominal wall; either one or 2 injections of 4 mg in sesame oil was given to each guinea-pig. The results are given in Table II. With this carcinogen most animals survived beyond 6 months. Tumour development was very much more erratic than with DEN. The tumours which were produced were mostly

TABLE II.—*Tumour Production with Methylcholanthrene*

| Strain of guinea-pig | MCA administration | No. of animals | No. of deaths before 6 months | Tumour incidence in survivors | Mean time for tumour development (months) |
|---|---|---|---|---|---|
| Hartley | 2 injections of 4 mg | 12 | 2/12 | 5/10 | 10·5 |
| | | 12 | 1/12 | 2/12 | 7 |
| | | 7 | 0/7 | 3/7 | 17 |
| | 1 injection of 4 mg | 4 | 0/4 | 2/4 | 7·5 |
| ICRF inbred | 1 injection of 4 mg | 5 | 1/5 | 4/4 | 10 |

sarcomata, almost all poorly differentiated and highly malignant. They included spindle cell sarcomata, liposarcomata and fibrosarcomata. Surprisingly, one was an adenocarcinoma which, taking its position into account, could have arisen from mammary gland tissue.

*B. The development of transplantable tumours*

(i) *Hepatoma.*—Three transplantable tumours have been developed from primary neoplastic livers. One was derived from a nodule of hepatocellular carcinoma in a Strain 13 animal and has been propagated *in vivo* for 29 months. It is relatively slow growing, infiltrates locally into muscle at the site of injection and if left for longer than 6 weeks tends to spread into the abdominal cavity and may metastasize to lungs. Two others were derived from primary hepatocellular carcinomata in inbred ICRF animals and have been propagated *in vivo* for 19 and 23 months respectively. Both these latter tumours grow locally on implantation and do not either infiltrate widely or metastasize spontaneously, but in some guinea-pigs from which the tumour had been removed surgically metastases to the lungs have subsequently occurred.

(ii) *Sarcoma.*—Three transplantable sarcomata have been developed and are being propagated in ICRF guinea-pigs. Two are liposarcomata which arose 8 months and 11 months respectively after single 4-mg injections of MCA; one is a fibrosarcoma which arose 10 months after a single 4-mg injection of MCA.

*C. Tissue culture of tumours and of normal liver tissue, and the development of established cell lines*

Approximately 20 primary liver tumours have been set up in monolayer tissue culture. It has been possible to obtain good primary cultures of all tumours attempted. Long-term continuous culture has not been attempted.

It has been possible to establish cultures from normal guinea-pig liver tissue using a single cell suspension obtained after perfusing the liver with collagenase and hyaluronidase in calcium-free Hanks' solution. The cells attach to the surface of Falcon plastic petri dishes and form monolayers in which cords and clusters of cells may be seen. Many of the cells are binucleate and for the first 4 or 5 days bear a close resemblance to hepatic parenchymal cells by their appearance in the light microscope and on electron microscopy. These appear to be in physiologically good condition and, like liver parenchymal cells examined under other conditions, respond to iontophoretically applied catecholamine with changes in membrane potential as measured with intracellular microelectrodes (Green, Dale and Haylett, 1972). Long-term culture has not been attempted.

A mesenteric metastatic nodule of a DEN hepatocellular carcinoma from a Hartley guinea-pig has been cultured and an established cell line obtained (VII : 3) which has been propagated in tissue culture for over 2 years. That the cells are malignant is proved by the fact that when injected into Hartley guinea-pigs as part of an immunization schedule, subcutaneous tumours developed in 5 of 20 animals given 6,000,000 cells or more. These transplanted tumours proved, on histological examination, to be cholangiocarcinomatous in type. This cell line has a 26-hour doubling time and its plating efficiency is 19%. Its chromosome count is 48 although there is some variation in chromosome number.

One of the transplantable tumours described above—a hepatocellular tumour of an inbred ICRF guinea-pig—has been successfully cultured and has been propagated *in vitro* for 13 months. The cells are clearly epithelial in type and they grow vigorously when transplanted back into guinea-pigs. With implantation of 100,000 cells there is 100% tumour incidence, good tumour production (approximately 50%) occurs with 10,000

cells and in some animals 1–5,000 cells have resulted in tumours. Doubling time is 26 hours and plating efficiency is 12%. The chromosome count is 96 and there is a considerable degree of aneuploidy.

### DISCUSSION

Because of the relative difficulty of tumour induction, comparatively little tumour work has been carried out on the guinea-pig compared with that performed on rats and mice. This difficulty led some workers to consider the guinea-pig as cancer resistant (Lombard, 1960) and others to search for anti-timour activity in guinea-pig serum (Kidd, 1953; Ainis *et al.*, 1958). Hartwell (1941, 1951) has reviewed the difficulties of early workers in inducing guinea-pig tumours, but Toth (1970) and Argus (1971) have reviewed later successes in this field.

Most of the tumours produced in the early studies were sarcomata, carcinomata being produced in only a few instances. No really consistent technique for producing carcinoma in the guinea-pig was available until the nitrosamines were used. Druckrey and Steinhoff (1962) reported 100% incidence of hepatic carcinoma in 11 guinea-pigs given DEN orally and since then there have been several successful studies using nitrosamines and other compounds (see Argus, 1971).

The most detailed work with DEN carcinogenesis in the guinea-pig has been carried out by Rapp and his co-workers at N.I.H. (Chrisler *et al.*, 1965; Rapp *et al.*, 1968). These workers obtained tumours in guinea-pigs given DEN orally, and reported not only the production of transplantable tumours but the development of ascites variants (Zbar *et al.*, 1969) and subsequent tissue culture of the ascitic tumours (Wepsic *et al.*, 1970). Since then, these cells have been used effectively in tumour immunology studies (Bernstein *et al.*, 1971; Churchill *et al.*, 1972; Wepsic *et al.*, 1971). Successful induction, transplantation and culture of a MCA liposarcoma in guinea-pigs was reported by Eilber, Holmes and Morton in 1971, the cultured tumour cells subsequently being used in immunotherapy experiments. Tissue culture of guinea-pig tumours has also been reported briefly by Laporte and Sillard (1967) and Leikina (1967).

Our results confirm that DEN given orally is a singularly successful carcinogen in guinea-pigs, particularly in Hartley guinea-pigs and the inbred ICRF animals, in which strains more animals survive the initial toxic effect of the chemical. MCA is effective but rather inconsistent in Hartley guinea-pigs but may be more consistent in ICRF animals. Tumours produced with both these agents are readily transplantable and short-term tissue culture of the primary tumours presents no great difficulty. Short-term tissue culture of normal guinea-pig liver tissue is also feasible for those immunological studies which require normal control tissues for comparison with cultures of primary tumours. We have also obtained two established cell lines, one of which (VII:3) has been propagated successfully and used for immunological studies in at least one other laboratory.

We are indebted to Dr P. M. Sutton of the Department of Morbid Anatomy, University College Hospital Medical School, for the assessment of the histopathology of the tumours. We would like to thank Miss C. Morris, Mrs J. Longcroft and Miss T. Billingsley for technical assistance.

This work was supported by a grant from the Cancer Research Campaign.

### REFERENCES

AINIS, H., KURTZ, H. M., KRAMER, P. I., WEIMER, H. E., RYAN, R. M. & JAMESON, E. (1958) *In vivo* and *in vitro* Studies of the Action of Guinea-pig Serum against the Ascites Form of the Murphy–Sturm Lymphosarcoma. *Cancer Res.*, **18**, 1309.

ARGUS, M. F. (1971) Susceptibility of the Guinea-pig to Chemical Carcinogenesis. *Cancer Res.*, **31**, 917.

BERNSTEIN, I. D., WEPSIC, H. T., ZBAR, B. & RAPP, H. J. (1971) Tumor Immunity: Impairment in Tumour-bearing Hosts. *J. natn. Cancer Inst.*, **46**, 873.

BERRY, M. N. & FRIEND, D. S. (1969) High Yield Preparation of Isolated Rat Liver Parenchymal Cells. *J. cell Biol.*, **43**, 506.

CHRISLER, C., RAPP, H. J., WEINTRAUB, R. M. & BORSOS, T. (1965) Forssman Antigen Content of Guinea-pig Hepatomas Induced by Diethylnitrosamine. *J. natn. Cancer Inst.*, **36**, 529.

CHURCHILL, W. H., ZBAR, B., BELLI, J. A. & DAVID, J. R. (1972) Detection of Cellular Immunity to Tumor Antigens of a Guinea-pig Hepatoma by Inhibition of Macrophage Migration. *J. natn. Cancer Inst.*, **48**, 541.

DRUCKREY, H. & STEINHOFF, D. (1962) Erzeugung von Leberkrebs im Meerschweinchen. *Naturwissenschaften*, **49**, 497.

EILBER, F. R., HOLMES, E. C. & MORTON, D. L. (1971) Immunotherapy Experiments with a Methylcholanthrene-induced Guinea-pig Liposarcoma. *J. natn. Cancer Inst.*, **46**, 803.

GREEN, R. D., DALE, M. M. & HAYLETT, D. G. (1972) Effect of Adrenergic Amines on the Membrane Potential of Guinea-pig Liver Parenchymal Cells in Short-term Tissue Culture. *Experientia*, **28**, 1073.

HARTWELL, J. L. (1941) *Survey of Components which have been Tested for Carcinogenic Activity.* Public Health Service Publ. 149. Washington: U.S. Govt. Printing Office.

HARTWELL, J. L. (1951) *Survey of Components which have been Tested for Carcinogenic Activity.* Public Health Service Publ. 149. Washington: U.S. Govt. Printing Office.

KIDD, J. G. (1953) Regression of Transplanted Lymphomas Induced *in vivo* by means of Normal Guinea-pig Serum. *J. exp. Med.*, **98**, 565.

LAPORTE, R. & SILLARD, R. (1967) Caracteres des cultures *in vitro* d'une tumeur transplantable du cobaye. *C.R. Acad. Sci., Paris*, **265**, 1575.

LEIKINA, F. I. (1967) A Strain of Guinea-pig Tumour Cells. *Vop. Virus*, **12**, 104.

LOMBARD, C. (1960) La cancero-resistance du Cobaye. *Bull. Ass. fr. Canc.*, **47**, 167.

MORTON, D. L., GOLDMAN, L. & WOOD, D. (1965) Tumour Specific Antigenicity of Methylcholanthrene and Dibenzanthracene Induced Sarcomas of Inbred Guinea-pigs. *Fedn. Proc.*, **24**, 684.

RAPP, H. J., CHURCHILL, W. H., KRONMAN, B. S., ROLLEY, R. T., HAMMOND, W. G. & BORSOS, T. (1968) Antigenicity of a New Diethylnitrosamine-induced Transplantable Guinea-pig Hepatoma: Pathology and Formation of an Ascites Variant. *J. natn. Cancer Inst.*, **41**, 1.

TOTH, B. (1970) Susceptibility of Guinea-pigs to Chemical Carcinogens: 7, 12-dimethylbenz (a) anthracene and Urethan. *Cancer Res.*, **30**, 2583.

WEPSIC, H. T., BERNSTEIN, I. D., ZBAR, B., BORSOS, T. & RAPP, H. J. (1971) Abrogation of Passively Transferred Tumour Immunity *in vivo* by Antigenically Related Tumour Cells. *J. natn. Cancer Inst.*, **46**, 195.

WEPSIC, T., KRONMAN, B. S., ZBAR, B., BORSOS, T. & RAPP, H. J. (1970) Immunotherapy of an Intramuscular Tumor in Strain 2 Guinea-pigs. *J. natn. Cancer Inst.*, **45**, 377.

ZBAR, B., WEPSIC, H. T., RAPP, H. J., WHANG-PENG, J. & BORSOS, T. (1969)) Transplantable Hepatomas Induced in Strain 2 Guinea-pigs by Diethylnitrosamine. *J. natn. Cancer Inst.*, **43**, 821.